# Supplementary material for: Structural mechanism underlying G protein family-specific regulation of G protein-gated inwardly rectifying potassium channel
Source: Nat Commun. 2019 May 1;10:2008. doi: 10.1038/s41467-019-10038-x (PMC6494913; doi:10.1038/s41467-019-10038-x)
Supplement: Supplementary file 1 — Supplementary Information [file 41467_2019_10038_MOESM1_ESM.pdf]

Supplementary information

Structural mechanism underlying G protein family-specific regulation of G protein-gated inwardly rectifying potassium channel

H. Kano *et al.*

Supplementary Figures

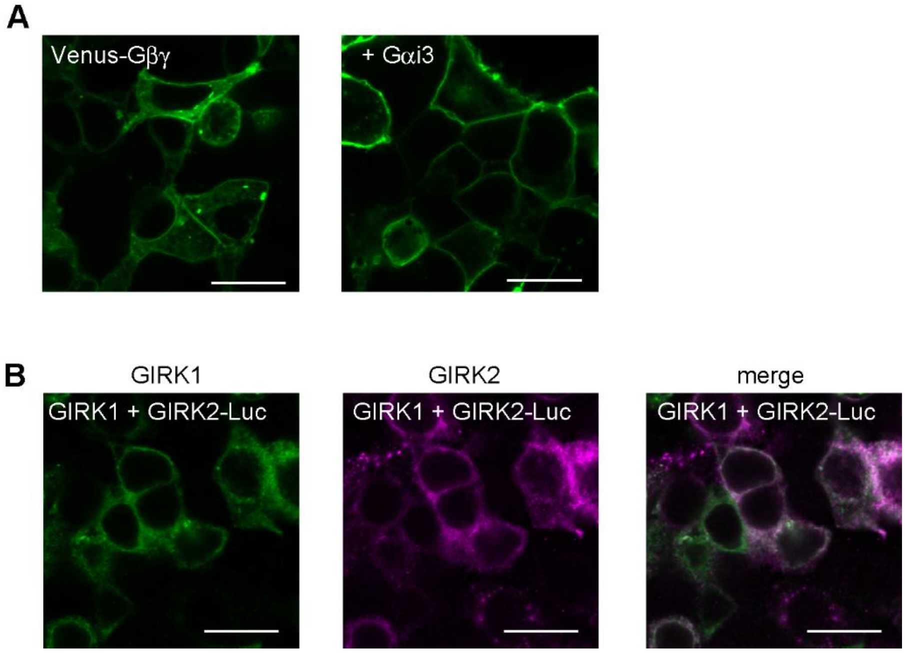

Supplementary Figure 1 Subcellular localization of Gαβγ and GIRK expressed in HEK293T cells. (A) Cells were transfected with Venus-Gβγ, with or without Gαi3. Localization of Venus-Gβγ was visualized by confocal fluorescence microscopy. (B) Cells were transfected with GIRK1 and GIRK2-Luc. Localizations of GIRK1 and GIRK2-Luc were visualized by immunostaining using anti-GIRK1 and anti-GIRK2 primary antibodies and fluorescent dye-conjugated secondary antibodies. Bar = 20 μm.

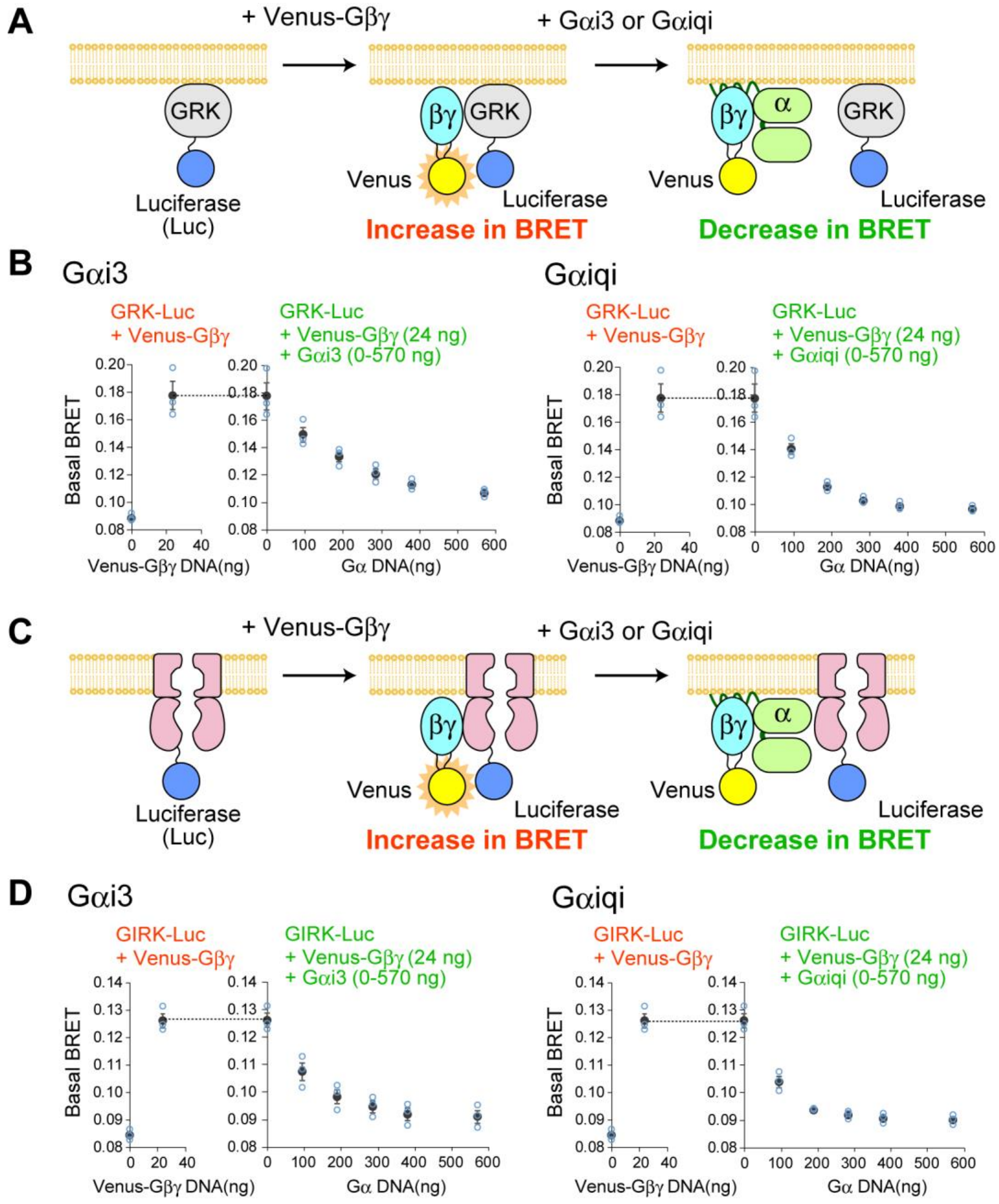

Supplementary Figure 2 BRET experiments observing the competitive binding of Gα for Venus-Gβγ. (A) Schematic representation of a competitive binding experiment observing the basal BRET between Gβγ and GRK. (B) Comparisons of the basal BRET values of cells expressing GRK-Luc, Venus-Gβγ, and Gαi3 (left) or Gαiqi (right). When the Gα expression levels were varied, the amounts of the transfected DNA encoding Gα were varied between 0 and 570 ng, while the amounts of the transfected

DNAs encoding GRK-Luc and Venus-Gβγ were fixed to 18 ng and 24 ng, respectively. (C) Schematic representation of a competitive binding experiment observing the basal BRET between Gβγ and GIRK. (D) Comparisons of the basal BRET values of cells expressing GIRK-Luc, Venus-Gβγ, and Gαi3 (left) or Gαiqi (right). When the Gα expression levels were varied, the amounts of the transfected DNA encoding Gα were varied between 0 and 570 ng, while the amounts of the transfected DNAs encoding GIRK1, GIRK2-Luc, and Venus-Gβγ were fixed to 18 ng, 18 ng, and 24 ng, respectively. Data are means ± SEM of 3 measurements taken from independently transfected cell batches. Source data are provided as a Source Data File.

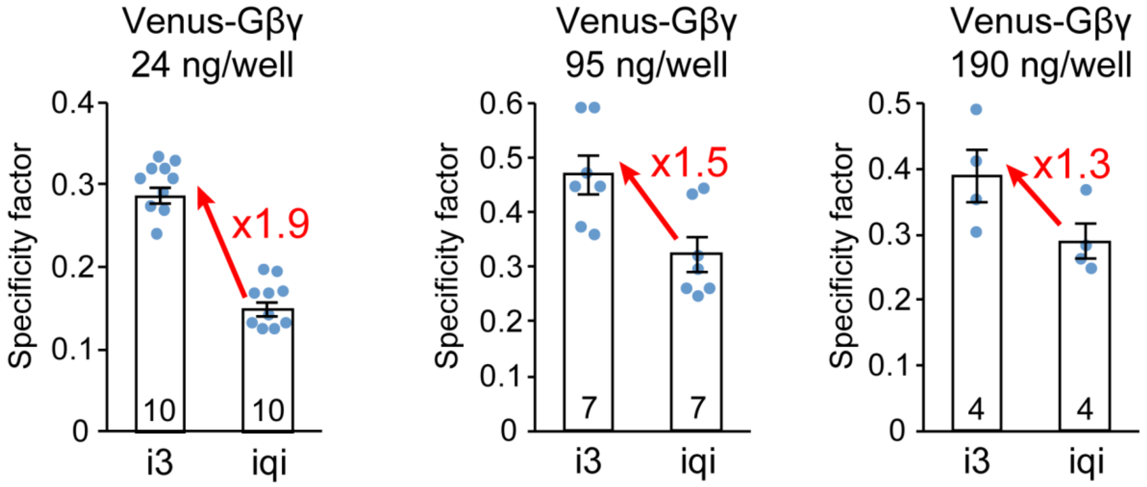

Supplementary Figure 3 Effects of the amount of expressed Gαβγ on the selectivity factors. The selectivity factors ($\Delta BRET_{GIRK}/\Delta BRET_{GRK}$ ratios) were measured in cells expressing different amounts of Gαi3βγ or Gαiqiβγ (n=4-10). For each of the indicated DNA amounts of Venus-Gβγ, the DNA amounts of Gα, GRK-Luc, GIRK1, and GIRK2-Luc were optimized and used (see Table 3). The ratios of the average values of i3 to those of iqi are shown in red. Data are means ± SEM. The number of measurements taken from independently transfected cell batches is indicated in the bar. Source data are provided as a Source Data File.

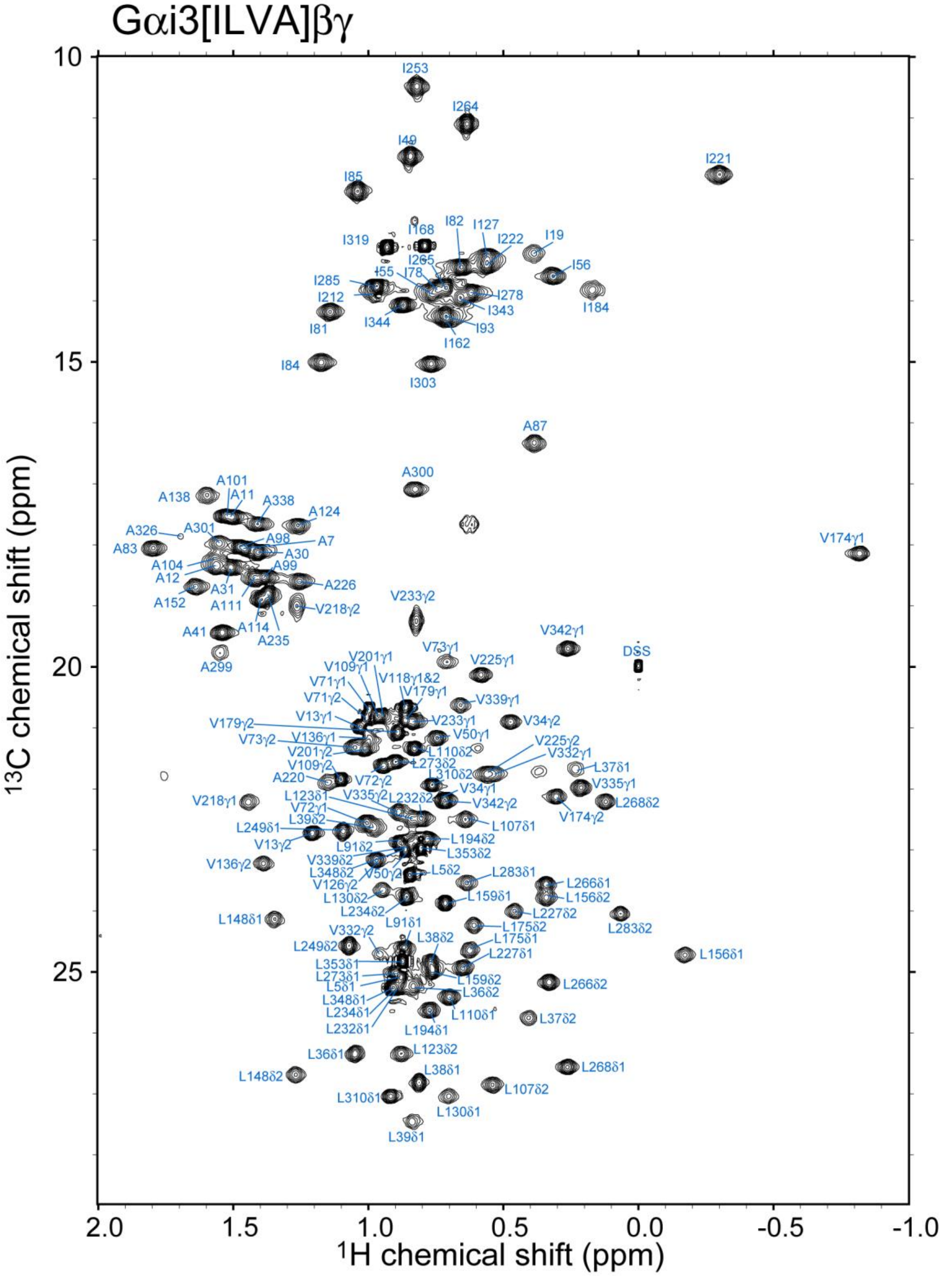

Supplementary Figure 4 **^{1}H-^{13}C HMQC spectrum of Gαi3βγ.** ^{1}H-^{13}C HMQC spectrum of {ul-[^{2}H, ^{15}N]; Alaβ, Ileδ1, Leuδ, Valγ-[$^{13}CH_3$]} Gαi3-[non-labeled]βγ and assignments of the methyl signals.

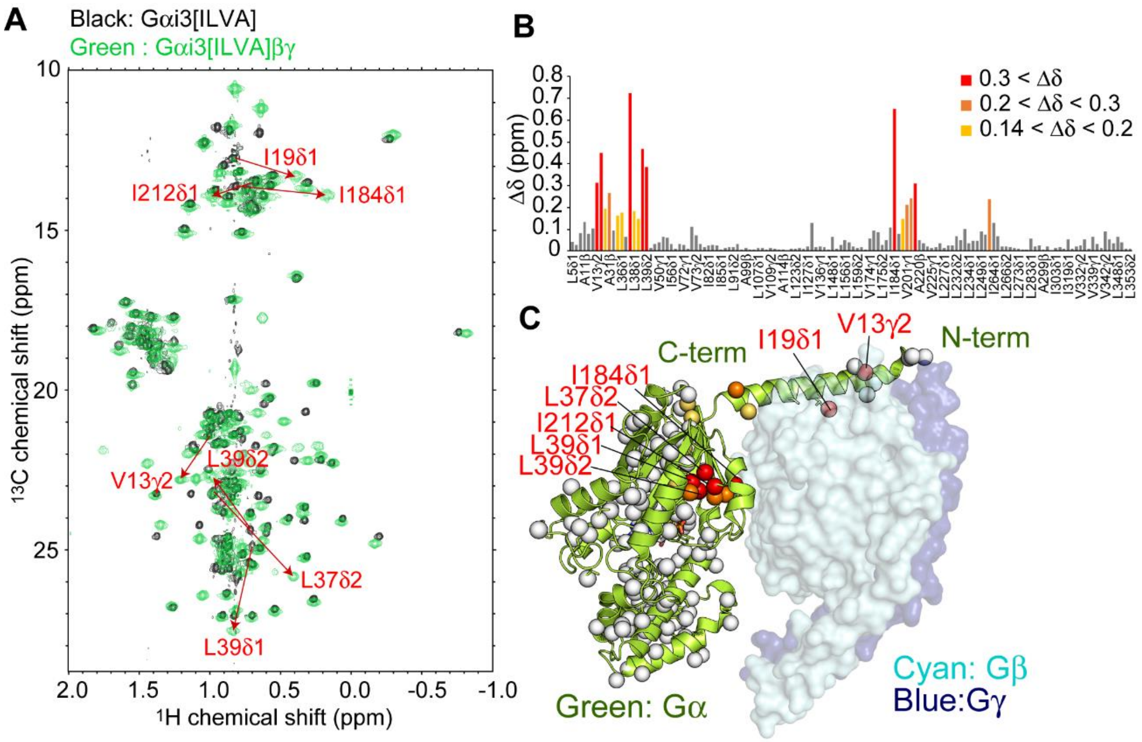

Supplementary Figure 5 Chemical shift differences between Gαi3(GDP) and Gαi3βγ. (A) Overlay of the ^{1}H-^{13}C HMQC spectra of Gαi3 [ILVA] in the GDP-bound form (black) and Gαi3[ILVA]βγ (green). The methyl groups with significant chemical shift differences are labeled. (B) Plot of the chemical shift differences between Gαi3(GDP) and Gαi3βγ. The averaged chemical shift differences are calculated by the equation, $\Delta\delta = [(\Delta\omega_H{}^2 + (\Delta\omega_C/5.9)^2]^{0.5}$. The methyl groups with chemical shift differences are colored according to their Δδ values. (C) The methyl groups with significant chemical shift differences are mapped on the crystal structure of Gαi1βγ (PDB ID: 1GP2). Methyl groups are shown as spheres and colored according to their Δδ values. Source data are provided as a Source Data File.

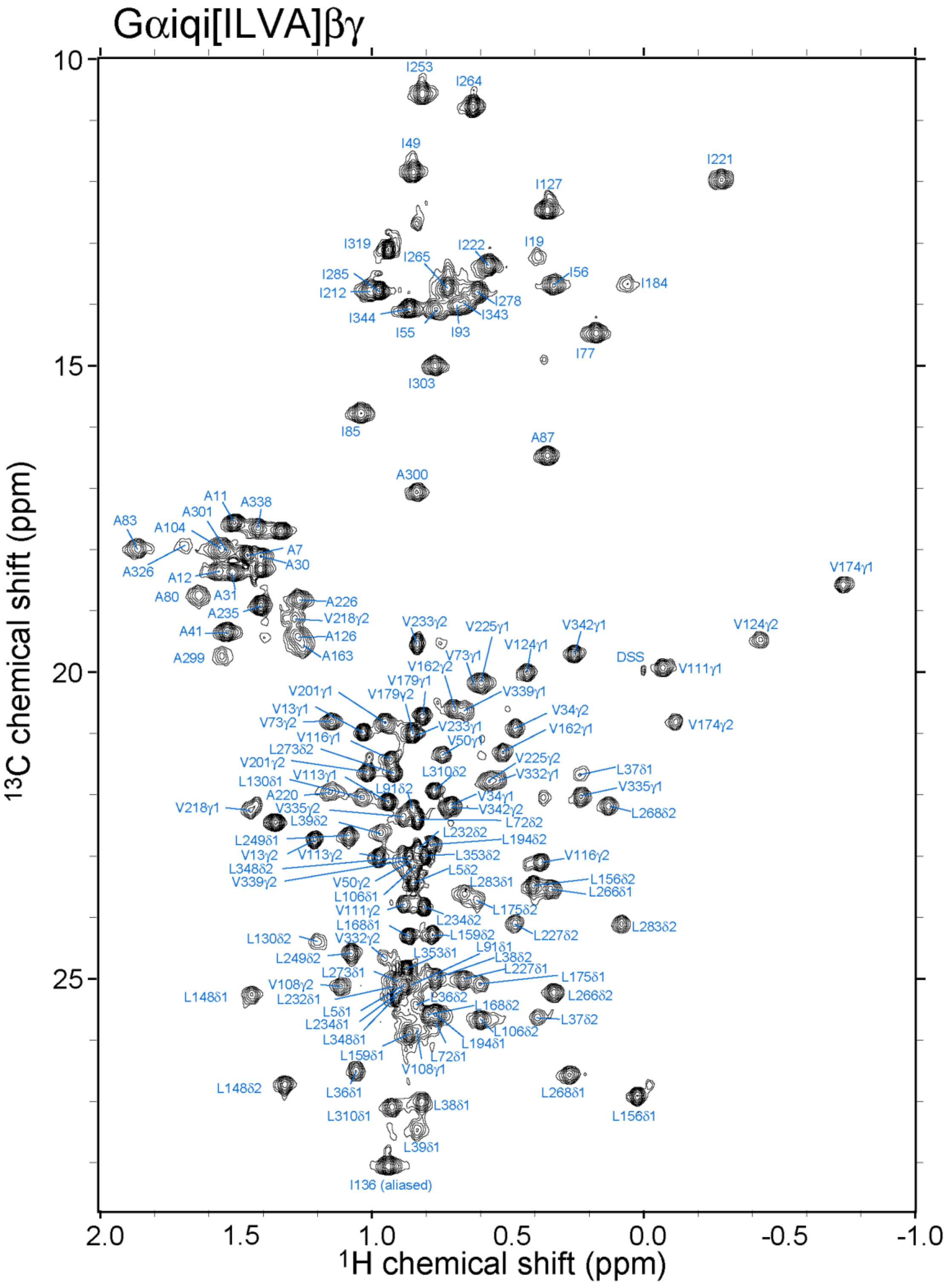

Supplementary Figure 6 **^1H-^{13}C HMQC spectrum of Gαiqiβγ.** ^1H-^{13}C HMQC spectrum of {ul-[^2H, ^{15}N]; Alaβ, Ileδ1, Leuδ, Valγ-[$^{13}CH_3$]} Gαiqi-[non-labeled]βγ and assignments of the methyl signals.

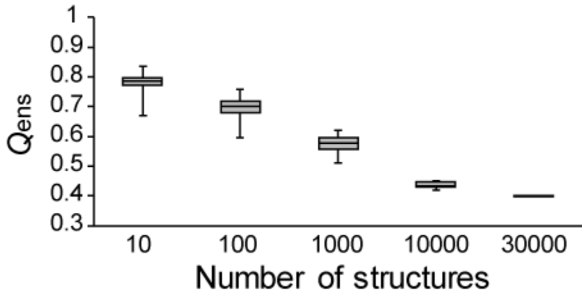

Supplementary Figure 7 Ensemble averaged Q-factors. Ensemble averaged Q-factors (Q_{ens}) representing agreement between the experimental and calculated PREs from the ensembles generated using 10, 100, 1,000, 10,000, and 30,000 structures. Numbers of samples are 1,000, 1,000, 100, 10, and 1, respectively. The boxes correspond to the 25‑75 percentile range, the middle line is the median, and the whiskers represent the full range from the minimum to the maximum. Source data are provided as a Source Data File.

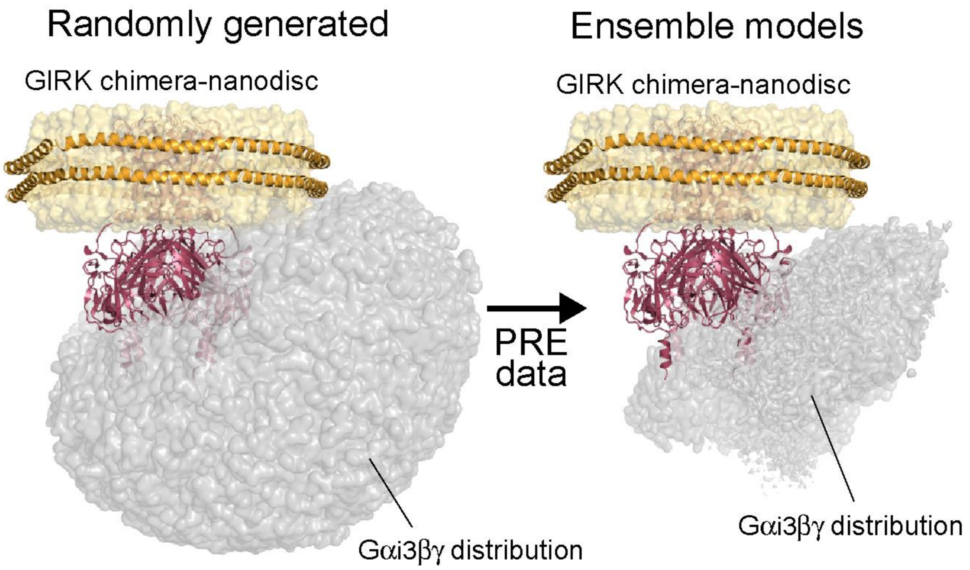

Supplementary Figure 8 Spatial distribution of Gαi3βγ. (Left) Spatial distribution of Gαi3βγ in randomly generated structures, in which the flexible region of the GIRK chimera was allowed to move freely. The distribution of Gαi3βγ in 1,000 structures sampled from 30,000 structures is shown as a gray surface. (Right) Spatial distribution of Gαi3βγ obtained from 10 ensemble calculations is displayed as an atomic probability density map, with a surface at the contour level of $\rho = 0.05$.

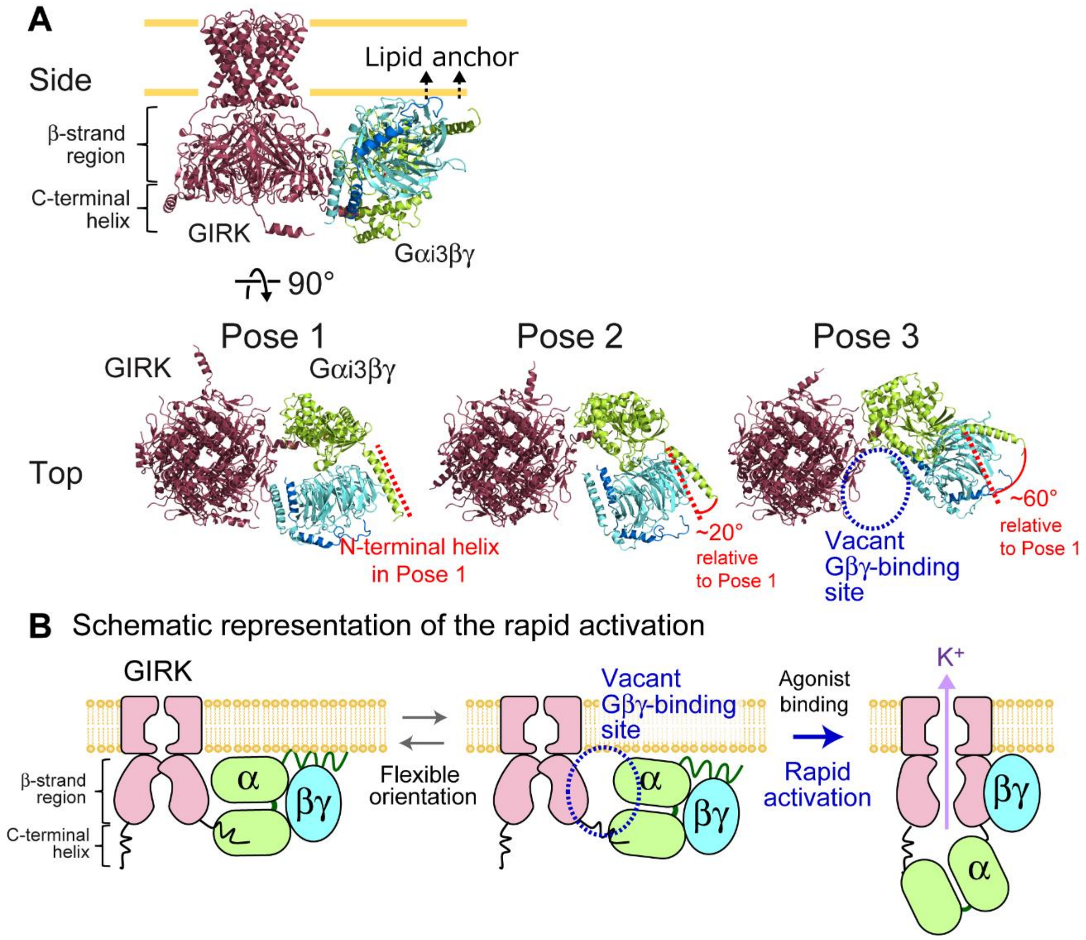

Supplementary Figure 9 Representative poses of the membrane-directed Gαi3βγ in complex with the GIRK chimera. (A) Side and top views of one structure (Pose 1), along with top views of two other structures (Poses 2 and 3) are displayed. The direction of the N-terminal helix in Pose 1 is marked as a red dotted line, and the relative angles of Gαi3βγ in Poses 2 and 3 to that in Pose 1 are shown. The open Gβγ-binding site in the Pose 3 orientation is shown as a blue dotted circle. (B) Schematic representation of the rapid activation of GIRK from the Gαi3βγ-GIRK complex. In the Gαi3βγ-GIRK complex, the relative orientation of Gαi3βγ toward the β-strand region of GIRK is rather flexible and the Gβγ-binding site on GIRK is transiently vacant, which enables the rapid activation of GIRK upon the agonist-binding to GPCR.

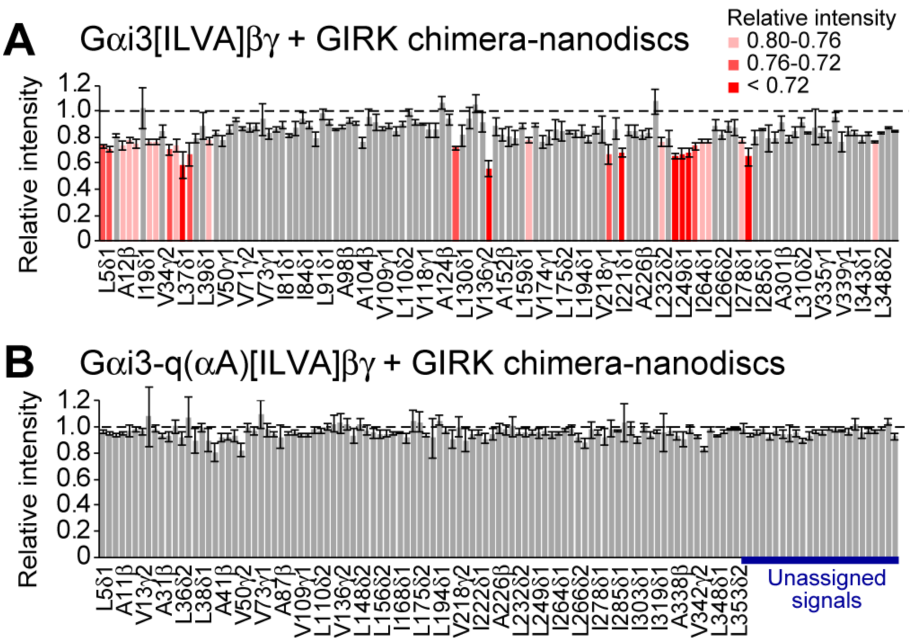

Supplementary Figure 10 NMR spectral changes of Gαi3-q(αA)[ILVA]βγ induced by the GIRK chimera-nanodiscs. Plots of relative intensities of Gαi3[ILVA]βγ (A) and Gαi3-q(αA) [ILVA]βγ (B) upon the addition of 2 eq. of the GIRK chimera-nanodiscs. The error bars are calculated based on the signal-to-noise ratios. Methyl groups with relative intensities lower than 0.80 are colored according to their values. The Gαi3[ILVA]βγ data are the same as those shown in Figure 2. Source data are provided as a Source Data File.

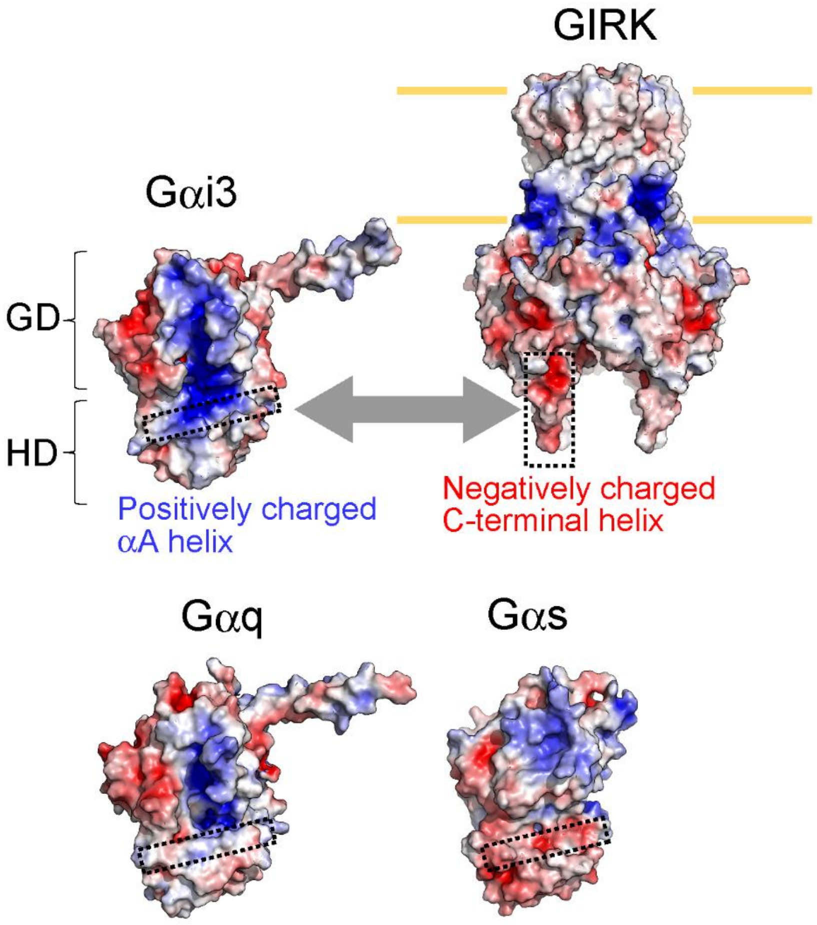

Supplementary Figure 11 The electrostatic potential surfaces of Gα and GIRK. The structures of Gαi3, Gαq, Gαs, and GIRK were derived from the crystal structures of Gαi1βγ (PDB ID: 1GP2) [1], Gαqβγ (PDB ID: 3AH8) [2], Gαs(GTPγS) (PDB ID: 1AZT) [3], and the GIRK chimera (PDB ID: 2QKS) [4], respectively. Electrostatic potential surfaces were calculated using the Adapted Poisson-Boltzmann Solver [5].

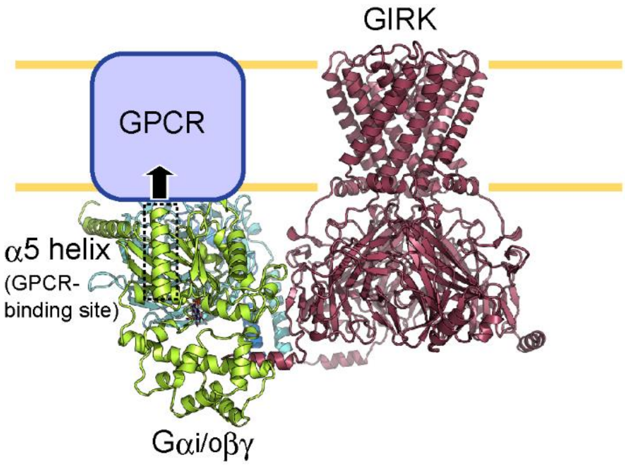

Supplementary Figure 12 The GPCR-binding site of Gα in the Gαi3βγ-GIRK complex. The major GPCR-binding site (α5 helix) of Gα is not involved in an interaction with GIRK. Our model structure of Gαi3βγ-GIRK is shown, and the α5 helix is marked with a dashed line. The GPCR in the membrane is depicted schematically.

Supplementary Tables

Supplementary Table 1 BRET values for BRET experiments using DOR inverse agonist

			(1) Ligand free	(2) Met-Enkephalin	(3) Met-Enkephalin + 10×excess ICI-174,864	(2)-(1) ΔBRET	n
DOR + GRK-Luc	no Gβγ	no Gα	0.0869±0.0004	0.0845±0.0004	0.0823±0.0004	-0.0024	3
	Venus-Gβγ	Gαi3	0.1265±0.0083	0.1735±0.0055	0.1236±0.0072	0.0470	3
		Gαiqi	0.1096±0.0055	0.1540±0.0097	0.1082±0.0061	0.0444	3
DOR + GIRK-Luc	no Gβγ	no Gα	0.0874±0.0008	0.0852±0.0008	0.0827±0.0005	-0.0022	3
	Venus-Gβγ	Gαi3	0.1006±0.0020	0.1101±0.0022	0.0987±0.0014	0.0095	3
		Gαiqi	0.0945±0.0009	0.1004±0.0022	0.0918±0.0013	0.0059	3

Supplementary Table 2 BRET values for BRET experiments using DOR antagonist

			(1) Ligand free	(2) Met-Enkephalin	(3) Met-Enkephalin + 10×excess Naloxone	(2)-(1) ΔBRET	n
DOR + GRK-Luc	no Gβγ	no Gα	0.0877±0.0001	0.0858±0.0001	0.0837±0.0002	-0.0019	3
	Venus-Gβγ	Gαi3	0.1005±0.0023	0.1426±0.0053	0.1046±0.0030	0.0421	10
		Gαiqi	0.0899±0.0013	0.1246±0.0041	0.0904±0.0016	0.0347	10
		Gαi3-q(αA)	0.0966±0.0021	0.1372±0.0046	0.0981±0.0019	0.0406	4
		Gαi3-q(αB)	0.1026±0.0031	0.1456±0.0096	0.1053±0.0040	0.0430	3
		Gαi3-q(αE)	0.1086±0.0038	0.1530±0.0065	0.1130±0.0046	0.0444	5
		* Gαqi5	0.0988	0.1008	0.0953	0.0020	1
DOR + GIRK-Luc	no Gβγ	no Gα	0.0885±0.0001	0.0863±0.0001	0.0837±0.0002	-0.0022	3
	Venus-Gβγ	Gαi3	0.0900±0.0015	0.1020±0.0022	0.0917±0.0015	0.0120	10
		Gαiqi	0.0850±0.0010	0.0902±0.0015	0.0834±0.0010	0.0052	10
		Gαi3-q(αA)	0.0917±0.0018	0.0982±0.0023	0.0887±0.0018	0.0065	4
		Gαi3-q(αB)	0.0900±0.0007	0.1003±0.0014	0.0917±0.0008	0.0103	3
		Gαi3-q(αE)	0.0948±0.0026	0.1057±0.0030	0.0961±0.0023	0.0109	5
		* Gαqi5	0.0874	0.0859	0.0844	-0.0015	1

* indicates the conditions where the activation of G proteins was not observed.

Supplementary Table 3 BRET values for BRET experiments using D2R

			(1) Ligand free	(2) Dopamine	(3) Dopamine + 10×excess Haloperidol	(2)-(1) ΔBRET	n
D2R + GRK-Luc	no Gβγ	no Gα	0.0881±0.0003	0.0863±0.0001	0.0833±0.0003	-0.0018	3
	Venus-Gβγ	Gαi3	0.1304±0.0035	0.1643±0.0057	0.1255±0.0029	0.0339	7
		Gαiqi	0.1215±0.0024	0.1487±0.0041	0.1192±0.0020	0.0272	7
		Gαqi5	0.1115±0.0009	0.1371±0.0032	0.1063±0.0009	0.0256	9
		Gαqiqi5	0.1255±0.0019	0.1638±0.0039	0.1219±0.0018	0.0383	7
D2R + GIRK-Luc	no Gβγ	no Gα	0.0885±0.0001	0.0863±0.0001	0.0834±0.0002	-0.0022	3
	Venus-Gβγ	Gαi3	0.1046±0.0019	0.1147±0.0020	0.1005±0.0014	0.0101	7
		Gαiqi	0.1023±0.0021	0.1066±0.0021	0.0972±0.0016	0.0043	7
		Gαqi5	0.0968±0.0006	0.0993±0.0016	0.0913±0.0005	0.0025	9
		Gαqiqi5	0.1033±0.0009	0.1122±0.0009	0.1010±0.0007	0.0089	7

Supplementary Table 4   Primer sequences for cloning

Primer name	Primer sequences (5’-3’)
GIRK1_Forward	ATGTCTGCACTCCGAAGGAAATTTG
GIRK1_Reverse	CTTTGTTATGTGAAGCGATCAGAGTTC
GIRK2_Forward	ATGGCCAAGCTGACAGAATCCATG
GIRK2_Reverse	CTAAACTTTGGATTCATTCTCCAGG
GRK3_Forward	CATGGCGGACCTGGAGGCCGTG
GRK3_Reverse	TCAGAGGCCGTTGCTGTTCCTGTG
DOR_Forward1	GGACGCCGGCAGCCATGGAA
DOR_Reverse1	GTACAACTAGGGAGGGGCGTCT
DOR_Forward2	AGCACAGTGGCGGCCACCATGGAACCGGCCCCCTC
DOR_Reverse2	AACGGGCCCTCTAGATCAGGCGGCAGCGCCACC
D2R_Forward	ATGGATCCACTGAATCTGTCCTGG
D2R_Reverse	TCAGCAGTGAAGGATCTTCAGGAAG
Galphai3_Forward	CGCCATGGGCTGCACGTTGAGC
Galphai3_Reverse	TCAATAAAGTCCACATTCCTTTAAGTTG
Galphaq_Forward	GAATGACTCTGGAGTCCATCATGG
Galphaq_Reverse	TTAGACCAGATTGTACTCCTTCAGG
Gbeta1_Forward	ATGAGTGAGCTTGACCAGTTACGG
Gbeta1_Reverse	TTAGTTCCAGATCTTGAGGAAGCTAC
Ggamma2_Forward	ATGGCCAGCAACAACACCGCC
Ggamma2_Reverse	TTAAAGGATGGCACAGAAAAACTTC
NLuc_Forward	CCACCATGGTCTTCACACTCGAAG
NLuc_Reverse	TTACGCCAGAATGCGTTCGCACAG

Supplementary Discussion

Contributions of the interaction formed with the nanodisc membrane

The lipid membrane also contributes to the formation of the complex, because the experimentally derived distribution of Gαi3βγ around the GIRK chimera-nanodiscs was highly skewed toward the membrane, as compared to the random distribution (Supplementary Figure 8), probably reflecting the interactions formed between the lipid molecules and Gαi3βγ. This observation is consistent with the result that the lipid-binding N-terminal region of Gαi3 showed a marked intensity reduction upon the interaction with the GIRK chimera-nanodiscs (Figure 2). Therefore, the experimentally derived ensemble is likely to represent the orientations of Gαi3βγ confined by the specific interactions with the GIRK chimera and the nanodisc membrane, confirming the importance of reconstituting the membrane environment for analyzing such physiological interactions.

Differences in binding mode between Gαi/o(GTP)-GIRK and Gαi/oβγ-GIRK

Although Gαi/oβγ essentially consists of the same proteins as active Gαi/o(GTP) and Gβγ, and the helical domain of Gα shares few structural differences between the inactive and the active states [6,7], the binding poses of Gαi/o with GIRK are quite different between the inactive and active states. Here, we discuss how the different binding poses can be adopted throughout the G protein cycling. Figure 5B shows the open-book views of the interacting sites on G proteins and the cytoplasmic region of GIRK. Gαi/oβγ interacts with GIRK predominantly through the helical domain (Figure 5B, green surface), whereas Gαi/o(GTP) interacts with GIRK mainly via the GTPase domain (Figure 5B, red surface) [8]. The GIRK-binding site on the GTPase domain [8] is not available in Gαi/oβγ,

because it is occupied by Gβγ, and thus the GTPase domain can only recognize GIRK in the form of Gαi/o(GTP). In addition to this, the structural differences in the N-terminal region of Gα between Gαi/oβγ and Gαi/o(GTP) also contribute to the different binding poses. In Gαi/oβγ, the N-terminal region forms a helix with a polybasic surface through the interaction with Gβγ, and the helix facilitates the membrane-anchoring to fix the orientation of Gαi/oβγ toward the membrane surface. In contrast, in Gαi/o(GTP), the N-terminal region is unfolded, which thereby allows the GTPase domain of Gαi/o(GTP) to be located farther from the membrane where it can interact with the C-terminal region of GIRK. It should be noted that the Gαi/o protein remains associated with GIRK throughout the G protein activation cycle, which would also contribute to the Gαi/o specificity.

Estimation of K_d for the Gαi3βγ-GIRK chimera-nanodiscs interaction

Based on our NMR observation of Gαi3βγ in the presence of the GIRK chimera-nanodiscs, the apparent K_d for the Gαi3βγ-GIRK chimera-nanodiscs interaction was calculated to be on the order of 10^{-4} M. Based on this K_d value, we then estimated the affinity of the Gαi3βγ-GIRK interaction under physiological conditions. The affinity of membrane proteins could be enhanced by the reduced dimensionality effects in the membrane; e.g., by 10^4-fold as compared to that measured in solution, where molecules freely translocate and tumble, as previously described in studies of the Gβγ-GIRK interaction [9,10]. Considering that Gαi3βγ could be partially localized to the membrane under our experimental conditions, the enhancement of the affinity under the membrane-delimited conditions is not expected to be as large as 10^4-fold. Thus, we estimated the K_d value for the Gαi3βγ-GIRK interaction to be in the sub-micromolar range on the cell membrane. To achieve the rapid activation of GIRK by Gβγ released from the activated

GPCRs, Gβγ needs to interact with GIRK without being perturbed by the Gαi/oβγ-GIRK interaction. Therefore, the affinity of the Gαi/oβγ-GIRK interaction is expected to be weaker than that of the Gβγ-GIRK interaction, which is represented by the half-maximal activation concentration for the Gβγ-induced GIRK activation (K_{act}) of 11 nM measured in electrophysiological experiments [9], and thus the estimated range for the Gαi/oβγ-GIRK interaction would be reasonable.

Functional implication of the flexibility in the relative orientation of Gαi/oβγ

Our structural ensemble model of the Gαi/oβγ-GIRK complex also suggested that there are multiple possible orientations even under the membrane-delimited conditions, in which the lipidation sites of Gαi/oβγ are directed toward the membrane (Supplementary Figure 9). No specific interaction between the structured β-strand region of GIRK and Gαi/oβγ was detected as site-specific PREs, and the interaction between the C-terminal helix of GIRK and the helical domain of Gαi/o was solely responsible for the functional coupling between GIRK and Gαi/oβγ in the BRET analyses. Therefore, we concluded that the variation in the relative orientation of Gαi/oβγ toward the β-strand region of GIRK would also exist under physiological conditions, and are not artefacts caused by the use of non-lipidated Gαi/oβγ. The variations in the relative orientations of Gαi/oβγ are in contrast to the fixed orientation of Gβγ toward GIRK, as demonstrated in our NMR analyses of the Gβγ-GIRK interaction and the crystal structure of the Gβγ-GIRK complex [10,11]. Since the Gβγ-binding site on GIRK is located on the structured β-strand region, the flexibility in the relative orientation of Gαi/oβγ allow Gβγ to access GIRK without being hindered by Gαi/oβγ, by making the Gβγ-binding site on GIRK transiently vacant. Thus, we assume that the inherent flexibility in the relative orientation

plays important roles in achieving the rapid activation of GIRK (Supplementary Figure 9). It should be noted that transient protein-protein interactions that take place on membrane, such as the Gαi3βγ-GIRK interaction, are usually difficult to detect by X-ray crystallographic and single-particle cryo-electron microscopic techniques, since they require the isolation of homogenous particles of stable complexes. In this sense, NMR analyses are quite beneficial to analyze the weak and transient protein-protein interactions with the K_d values on the order of the micromolar to millimolar range. Our results suggest that such transient interactions on the cell membrane actually play important roles in determining the specificity of intracellular signaling.

Suppression of the GIRK current by phospholipase C

The Gαi/o-specific activation of GIRK has also been explained by the alternative hypothesis that the Gαq-induced activation of phospholipase C (PLC) inhibits the GIRK current by decreasing the amount of membrane-localized PIP_2, which is an essential co-factor for the opening of GIRK [17]. Although the PIP_2 depletion reportedly contributes to the slow rundown of the GIRK current [18,19], the receptor-mediated activation of GIRK usually occurs on the order of 10^{-1}-10^{0} second [20], which is faster than the PLC hydrolysis reaction rates, on the order of tens of seconds [21]. Thus, the mechanism does not fully explain the fact that the rapid activation of GIRK is suppressed upon the stimulation of Gq-coupled receptors. Our hypothesis that the formation of the Gαi/oβγ-GIRK complex predetermines the signaling pathway can explain this experimental observation, which is also supported by the cell-based BRET experiments, in which the binding of Gβγ to GIRK itself is deficient upon the activation of Gq, probably due to the inability of Gq to pre-form the Gαβγ-GIRK complex. This model also explains the previous observations that

the activation of GIRK is invoked exclusively by Gi/o-coupled GPCRs, not by GPCRs coupled to other Gα families such as Gs [22].

Supplementary References

1. Wall, M. A. *et al.* The structure of the G protein heterotrimer $G_{i\alpha1}\beta_1\gamma_2$. *Cell* **83,** 1047–1058 (1995).
2. Nishimura, A. *et al.* Structural basis for the specific inhibition of heterotrimeric G_q protein by a small molecule. *Proc. Natl. Acad. Sci. U. S. A.* **107,** 13666–71 (2010).
3. Sunahara, R. K., Tesmer, J. J., Gilman, A. G. & Sprang, S. R. Crystal structure of the adenylyl cyclase activator $G_{s\alpha}$. *Science* **278,** 1943–7 (1997).
4. Nishida, M., Cadene, M., Chait, B. T. & MacKinnon, R. Crystal structure of a Kir3.1-prokaryotic Kir channel chimera. *EMBO J.* **26,** 4005–4015 (2007).
5. Jurrus, E. *et al.* Improvements to the APBS biomolecular solvation software suite. *Protein Sci.* **27,** 112–128 (2018).
6. Oldham, W. M. & Hamm, H. E. Heterotrimeric G protein activation by G-protein-coupled receptors. *Nat. Rev. Mol. Cell Biol.* **9,** 60–71 (2008).
7. Sondek, J., Bohm, A., Lambright, D. G., Hamm, H. E. & Sigler, P. B. Crystal structure of a G_A protein βγ dimer at 2.1 Å resolution. *Nature* **379,** 369–374 (1996).
8. Mase, Y., Yokogawa, M., Osawa, M. & Shimada, I. Structural basis for modulation of gating property of G protein-gated inwardly rectifying potassium ion channel (GIRK) by i/o-family G protein α subunit ($G\alpha_{i/o}$). *J. Biol. Chem.* **287,**

19537–19549 (2012).

9. Wickman, K. D. *et al.* Recombinant G-protein βγ-subunits activate the muscarinic-gated atrial potassium channel. *Nature* **368,** 255–257 (1994).

10. Yokogawa, M., Osawa, M., Takeuchi, K., Mase, Y. & Shimada, I. NMR analyses of the Gβγ binding and conformational rearrangements of the cytoplasmic pore of G protein-activated inwardly rectifying potassium channel 1 (GIRK1). *J. Biol. Chem.* **286,** 2215–2223 (2011).

11. Whorton, M. R. & MacKinnon, R. X-ray structure of the mammalian GIRK2-βγ G-protein complex. *Nature* **498,** 190–197 (2013).

12. Fowler, C. E., Aryal, P., Suen, K. F. & Slesinger, P. A. Evidence for association of $GABA_B$ receptors with Kir3 channels and regulators of G protein signalling (RGS4) proteins. *J. Physiol.* **580,** 51–65 (2007).

13. Richard-Lalonde, M. *et al.* Conformational Dynamics of Kir3.1/Kir3.2 Channel Activation Via δ-Opioid Receptors. *Mol. Pharmacol.* **83,** 416–428 (2013).

14. Tateyama, M. & Kubo, Y. Gi/o-coupled muscarinic receptors co-localize with GIRK channel for efficient channel activation. *PLoS One* **13,** e0204447 (2018).

15. Rasmussen, S. G. F. *et al.* Crystal structure of the β_2 adrenergic receptor-Gs protein complex. *Nature* **477,** 549–557 (2011).

16. Touhara, K. K. & MacKinnon, R. Molecular basis of signaling specificity between GIRK channels and GPCRs. *Elife* **7,** 399–404 (2018).

17. Wang, W., Whorton, M. R. & MacKinnon, R. Quantitative analysis of mammalian GIRK2 channel regulation by G proteins, the signaling lipid PIP2 and Na+ in a reconstituted system. *Elife* **3,** e03671 (2014).

18. Kobrinsky, E., Mirshahi, T., Zhang, H., Jin, T. & Logothetis, D. E. Receptor-

mediated hydrolysis of plasma membrane messenger PIP_2 leads to K^+-current desensitization. *Nat. Cell Biol.* **2,** 507–514 (2000).

19. Lei, Q., Talley, E. M. & Bayliss, D. A. Receptor-mediated Inhibition of G Protein-coupled Inwardly Rectifying Potassium Channels Involves $G\alpha_q$ Family Subunits, Phospholipase C, and a Readily Diffusible Messenger. *J. Biol. Chem.* **276,** 16720–16730 (2001).
20. Doupnik, C. A., Jaén, C. & Zhang, Q. Measuring the modulatory effects of RGS proteins on GIRK channels. *Methods Enzymol.* **389,** 131–154 (2004).
21. Van der Wal, J., Habets, R., Várnai, P., Balla, T. & Jalink, K. Monitoring Agonist-induced Phospholipase C Activation in Live Cells by Fluorescence Resonance Energy Transfer. *J. Biol. Chem.* **276,** 15337–15344 (2001).
22. Leaney, J. L., Milligan, G. & Tinker, A. The G protein α subunit has a key role in determining the specificity of coupling to, but not the activation of, G protein-gated inwardly rectifying K^+ channels. *J. Biol. Chem.* **275,** 921–929 (2000).
