# Supplementary material for: Structural mechanism underlying G protein family-specific regulation of G protein-gated inwardly rectifying potassium channel
Source: Nat Commun. 2019 May 1;10:2008. doi: 10.1038/s41467-019-10038-x (PMC6494913; doi:10.1038/s41467-019-10038-x)
Supplement: Supplementary file 3 — Reporting Summary [file 41467_2019_10038_MOESM3_ESM.pdf]

nature research

Corresponding author(s): Ichio Shimada

Last updated by author(s): Apr 2, 2019

Reporting Summary

Nature Research wishes to improve the reproducibility of the work that we publish. This form provides structure for consistency and transparency in reporting. For further information on Nature Research policies, see Authors & Referees and the Editorial Policy Checklist.

Statistics

For all statistical analyses, confirm that the following items are present in the figure legend, table legend, main text, or Methods section.

n/a	Confirmed	
☐	☒	The exact sample size (*n*) for each experimental group/condition, given as a discrete number and unit of measurement
☐	☒	A statement on whether measurements were taken from distinct samples or whether the same sample was measured repeatedly
☐	☒	The statistical test(s) used AND whether they are one- or two-sided<br>*Only common tests should be described solely by name; describe more complex techniques in the Methods section.*
☒	☐	A description of all covariates tested
☐	☒	A description of any assumptions or corrections, such as tests of normality and adjustment for multiple comparisons
☐	☒	A full description of the statistical parameters including central tendency (e.g. means) or other basic estimates (e.g. regression coefficient) AND variation (e.g. standard deviation) or associated estimates of uncertainty (e.g. confidence intervals)
☐	☒	For null hypothesis testing, the test statistic (e.g. *F*, *t*, *r*) with confidence intervals, effect sizes, degrees of freedom and *P* value noted<br>*Give P values as exact values whenever suitable.*
☒	☐	For Bayesian analysis, information on the choice of priors and Markov chain Monte Carlo settings
☒	☐	For hierarchical and complex designs, identification of the appropriate level for tests and full reporting of outcomes
☒	☐	Estimates of effect sizes (e.g. Cohen's *d*, Pearson's *r*), indicating how they were calculated

Our web collection on statistics for biologists contains articles on many of the points above.

Software and code

Policy information about availability of computer code

Data collection	All NMR spectra were processed by the Bruker TopSpin 2.1 or 3.1 software.<br>Homology models were built using the SWISS-MODEL SERVER (https://swissmodel.expasy.org).<br>The structures of the nanodiscs were built using the CHARMM-GUI Nanodisc Builder (http://www.charmm-gui.org/).<br>Structural calculations were performed using XPLOR-NIH version 2.48. Codes for structural calculations are available from the corresponding author upon request.
Data analysis	The NMR data were analyzed using Sparky 3.<br>The analysis of the structural models was performed using R language 3.4.3. Codes are available from the corresponding author upon request.<br>The structures were rendered using PyMOL 2.0.7.

For manuscripts utilizing custom algorithms or software that are central to the research but not yet described in published literature, software must be made available to editors/reviewers. We strongly encourage code deposition in a community repository (e.g. GitHub). See the Nature Research guidelines for submitting code & software for further information.

Data

Policy information about availability of data

All manuscripts must include a data availability statement. This statement should provide the following information, where applicable:

- Accession codes, unique identifiers, or web links for publicly available datasets
- A list of figures that have associated raw data
- A description of any restrictions on data availability

The manuscript include a data availability statement.

Field-specific reporting

Please select the one below that is the best fit for your research. If you are not sure, read the appropriate sections before making your selection.

☒ Life sciences ☐ Behavioural & social sciences ☐ Ecological, evolutionary & environmental sciences

For a reference copy of the document with all sections, see nature.com/documents/nr-reporting-summary-flat.pdf

Life sciences study design

All studies must disclose on these points even when the disclosure is negative.

Sample size	No statistical methods were used to predetermine sample sizes.
Data exclusions	The data were not excluded from the analysis.
Replication	All attempts at replication were successful.
Randomization	Randomization method was not utilized.
Blinding	Investigator was not blinded to the group allocation.

Reporting for specific materials, systems and methods

We require information from authors about some types of materials, experimental systems and methods used in many studies. Here, indicate whether each material, system or method listed is relevant to your study. If you are not sure if a list item applies to your research, read the appropriate section before selecting a response.

Materials & experimental systems

n/a	Involved in the study	
☐	☒	Antibodies
☐	☒	Eukaryotic cell lines
☒	☐	Palaeontology
☒	☐	Animals and other organisms
☒	☐	Human research participants
☒	☐	Clinical data

Methods

n/a	Involved in the study	
☒	☐	ChIP-seq
☒	☐	Flow cytometry
☒	☐	MRI-based neuroimaging

Antibodies

Antibodies used	1. Rabbit polyclonal to GIRK1 (Abcam ab96168)<br>2. Goat polyclonal to GIRK2 (Abcam ab65096)<br>3. Donkey polyclonal Secondary Antibody to Rabbit IgG - H&L (Alexa Fluor® 488) (Abcam ab150073)<br>4. Donkey polyclonal Secondary Antibody to Goat IgG - H&L (Alexa Fluor® 647) (Abcam ab150131)
Validation	1. Validations were performed by the manufacturer using A431, H1299, HeLa whole cells lysate, and DU145 xenograft as positive controls.<br>2. Validations were performed by the manufacturer using Human Brain (Substantia Nigra) lysate as a positive control.

Eukaryotic cell lines

Policy information about cell lines

Cell line source(s)	293T (ECACC 12022001), Sf9 (Thermo Fisher Scientific), expresSF+ (Protein Sciences)
Authentication	The 293T cell line was authenticated by ECACC using STR-PCR.<br>The Sf9 and expresSF+ cell lines were not authenticated.
Mycoplasma contamination	The 293T cell line tested negative for mycoplasma contamination by MycoAlert Mycoplasma Detection Kit (Lonza).<br>The Sf9 and expresSF+ cell lines were not tested for mycoplasma contamination.
Commonly misidentified lines (See ICLAC register)	No commonly misidentified cell lines were used.
